# A Comparative Study of Causality Detection Methods in Root Cause Diagnosis: From Industrial Processes to Brain Networks

**DOI:** 10.3390/s24154908

**Published:** 2024-07-29

**Authors:** Sun Zhou, He Cai, Huazhen Chen, Lishan Ye

**Affiliations:** 1 Department of Automation, Xiamen University, Xiamen 361102, China; caihe@stu.xmu.edu.cn; 2 School of Sociology and Anthropology, Xiamen University, Xiamen 361005, China; chenhuazhen@stu.xmu.edu.cn; 3 Institute of Brain and Cognitive Sciences, Tsinghua University, Beijing 100084, China

**Keywords:** causality detection, causal inference, root cause analysis, brain connectivity network, Granger causality

## Abstract

Abstracting causal knowledge from process measurements has become an appealing topic for decades, especially for fault root cause analysis (RCA) based on signals recorded by multiple sensors in a complex system. Although many causality detection methods have been developed and applied in different fields, some research communities may have an idiosyncratic implementation of their preferred methods, with limited accessibility to the wider community. Targeting interested experimental researchers and engineers, this paper provides a comprehensive comparison of data-based causality detection methods in root cause diagnosis across two distinct domains. We provide a possible taxonomy of those methods followed by descriptions of the main motivations of those concepts. Of the two cases we investigated, one is a root cause diagnosis of plant-wide oscillations in an industrial process, while the other is the localization of the epileptogenic focus in a human brain network where the connectivity pattern is transient and even more complex. Considering the differences in various causality detection methods, we designed several sets of experiments so that for each case, a total of 11 methods could be appropriately compared under a unified and reasonable evaluation framework. In each case, these methods were implemented separately and in a standard way to infer causal interactions among multiple variables to thus establish the causal network for RCA. From the cross-domain investigation, several findings are presented along with insights into them, including an interpretative pitfall that warrants caution.

## 1. Introduction

Abstracting causal knowledge from process measurements has been an important and challenging research topic for decades. Causality detection seeks to capture the cause-and-effect interaction between variables. Compared to nondirected relation metrics such as correlation, coherence, etc., it establishes a statistical directed relation that considers the temporal structure in the data. Although many mathematical conceptual problems of causality are still being debated [1], various causality analysis methods have sprung up in different domains such as process industry [2,3], computational neuroscience [4,5], economics [6], environmental ecology [7,8], physics [1,9], and much else. Especially for fault root cause or root causes analysis (RCA) in a complex system, causality detection has become attractive in practice owing to the large amount of available data and low budget requirement and has already led to important insights.

Once the data, which are usually measured from multiple sensors over a long time, have been collected, the challenge for the experimental researcher (or engineer) is to find appropriate ways to detect and quantify causal interactions across multiple variables and to provide a valid interpretation of the findings. This is challenging for several reasons.

First, in the literature on causality analysis, comparative study across distinct fields is rare. Although there are many publications that provide a review or survey [10], they either discuss mathematically the fundamental theories of causal inference or limit the research within a specific field. Studies in the literature from a mathematical perspective include the comprehensive treatments of Pearl (2009) [11], Spirtes et al. (2000) [12], Imbens and Rubin (2015) [13], and Peters et al. (2017) [14]. In the industrial process monitoring area, various causality detection methods applied to diagnosis of the root cause of different types of faults have been well reviewed in publications [10,15]. In the neuroscience domain, many studies in the literature have provided comprehensive treatments of causal inference approaches that are applicable in the analysis of brain functional connectivity or effective connectivity networks [16,17,18,19]. It is worth pointing out that some research communities may have an idiosyncratic implementation of their preferred causality measures, with limited accessibility to the wider research community. In fact, as comparative studies of causality detection methods across fields are rare, it is challenging for an experimental researcher or an engineer to be aware of which methods may not be applicable in which domains as well as which methods, if any, may be realistically effective across different domains.

Second, selecting and justifying which method to use is often difficult. Studies in the literature provide many methods to quantify interactions, often described with a great deal of technical details. Some methods such as Granger causality (GC) are based on a rigorous statistical theory of stochastic processes, while others, such as convergent cross mapping (CCM), are based on chaos theory. Each method has its own presuppositions, advantages, and limitations, as well as its own adherents and opponents.

Finally, both the applicability of some particular causal inference methods and their comparison with the others are complicated. For the same method, in different studies in the literature the cases used may be different or the experimental conditions and settings for the same case may be heterogeneous.

This work provides a comprehensive comparison of data-based causality detection methods with two RCA problems from distinct domains. Targeting interested experimental researchers or engineers, this article intends to help increase the awareness of assumptions and the scope of applicability of different methods, as well as the impact of the systems through which the fault propagates in the results of the method. Of the two RCA problems, one is fault or disturbance root cause tracking in industrial processes, while the other is an estimation of the effective connectivity between neuronal populations in the human brain, which is even more complex. Both problems are caused by a local fault or local abnormal activity propagating through the system or network, which eventually leads to a widespread fault or failure.

A RCA problem in industrial processes

In the last three decades, a lot of work has been performed on fault detection and diagnosis, and various techniques have been developed, ranging from model-based approaches (including observer-based [20] and structural graphs [21]) to data-driven approaches (including classifiers [22], pattern recognition, and neural networks [23]).

In modern industrial processes, plants are usually composed of multiple control loops and numerous interconnected devices. Their complex connections may lead to the propagation of faults through the information and material flow pathways. Locating the root cause in certain abnormal situations is very important in ensuring safety. Existing RCA methodologies in the process monitoring field can be viewed as three types: knowledge-based approaches [24,25], data-based approaches, and integrations of both [26]. Data-based approaches have received considerable attention due to the large amount of data in computerized processes. This type can be further classified into several categories, namely, time series causality analysis, probabilistic graphical models, machine learning and other data-based approaches. This work focuses on the time series causality analysis strategy.

Shao et al. proposed the Copula-based GC [27], which extends the GC, to analyze the causal relations between nonlinearly related process observations of plant-wide oscillation. He et al. [28] also presented a nonlinear extension of a GC-based method. For RCA of triggered alarms in chemical processes, a causality detection solution based on a multivariate GC (MVGC) test and Gaussian process regression (GPR) was put forward by Chen et al., where GPR was used to address the problem of GC measures for nonstationary and nonlinearly related time series [29]. An advantage of GPR-based GC is that in handling nonstationary observations, it preserves the trends in the time series, while in the most-utilized methods, such as the removal of the trend component by some kind of decomposition and first-order difference method [30], missing the trends is a possible problem. Some algorithms were developed and shown to outperform GC in some cases and were able to deal with large-scale networks, such as optimal causation entropy (OCE) [31], which was proposed for computational and data-efficient algorithms for causal network inference, and the PCMCI [32], with a conditional mutual information (MCI) test to reduce autocorrelation and to control false-positive rates.

In Duan et al. [33], the concept of transfer 0-entropy was formed, which does not assume a well-defined probability distribution for process observations. With consideration of the trends of process data, a trend transfer entropy (TTE) method was developed to represent trend causality rather than value causality between the variables [34]. A symbolic, dynamic-based, normalized transfer entropy (SDNTE) method was proposed in Rashidi et al. [35] to facilitate an efficient real-time RCA. Using the concept of information granulation for data compression, a transfer entropy (TE)-based causality detection method was developed by Zhang et al. [36] to address the problem of computational complexity of TE in high-dimensional embedded spaces.

B Source analysis in brain networks

The human brain is a complex system with underlying mechanisms that remain mostly unknown. When it comes to locating the dysfunctional source region in the brain in a neurological disorder, the situation is more complicated. One theory claims that the brain can dynamically coordinate the flow of information by changing the intensity, pattern, or frequency with which different brain regions or neuronal populations engage in oscillatory synchrony. By using the appropriate causality detection measure, the direction and strength of information flow between oscillatory neuronal signals, called effective connectivity, can be quantified to estimate the causal influence one neuronal population exerts on another.

A comprehensive characterization of brain networks can be constructed by means of estimating brain effective connectivity. Such a characterization not only produces a wealth of accounts of normal cognitive processes [37,38] but also helps to gain new insights into the role that brain network dysfunction plays in neurological disorders, such as epilepsy, autism, depression, schizophrenia, and post-concussion syndrome [39].

A variety of neuroimaging techniques have provided ways to measure connectivity between brain regions, including functional magnetic resonance imaging (fMRI), positron emission computed tomography (PET), functional near-infrared spectroscopy (fNIRS), electroencephalogram (EEG), and magnetoencephalogram (MEG). They can be obtained with invasive or noninvasive recording techniques in an experimental manipulation or in a task-free context.

Most interactions between the brain regions are nonlinear [40]. However, a limitation of GC measures is that they assume the process to be wide-sense stationary and use a linear predictor with an autoregressive (AR) model. Some nonlinear GC methods proposed in [41,42] help to remediate the linear prediction of GC measures. To relax the stationarity assumption to provide time-varying effective connectivity causal analysis based on GC, several methods have been developed. Al-Ezzi et al. utilized the phase slope index (PSI) to estimate the effective connectivity network from EEG data [43]. Pagnotta et al. [44] and Pascucci et al. [45] modeled the dynamic changes in the brain network with a time-varying multivariate AR (MVAR) model using adaptive Kalman filters to provide estimates of the effective connectivity with time in accordance with known physiology. The works of Jiang et al. [46] and Yang et al. [47] used an extension of directed transfer function (DTF), called adaptive DTF (ADTF), based on a time-varying MVAR model to construct the effective connectivity networks during a seizure. In [48], a data-driven and model-based likelihood estimator is used to estimate the causal interactions between the electrodes.

Table 1 outlines the distinctions between both systems that we selected for this study from distinct domains.

The main contributions of this article are outlined as follows: (i) To the best of our knowledge, this article makes the first comparative study of data-based causality detection methods across distinct domains. Two RCA problems in distinct complex systems were investigated. (ii) Considering the differences of various causality detection methods, we designed several sets of experiments so that for each case, a total of 11 methods could be appropriately compared under a unified and reasonable evaluation framework. (iii) From the cross-domain comparison, several findings are presented along with insights into them, including an interpretative pitfall that warrants caution.

The remainder of this paper is organized as follows. Section 2 presents descriptions of the two problems. In Section 3, a possible taxonomy of time series causality analysis methods is provided, followed by an overview of those concepts. Then our experimental design, implementation of those methods, and the results are presented. Section 4 makes a comprehensive discussion based on the results and points out some issues that warrant caution, with suggestions. Also, a brief introduction with technical details to the popular or up-to-date causality detection methods based on time series can be found in Appendix A.

## 2. Materials

Two RCA problems from distinct research communities are studied in this work by focusing on the causal effect estimation from a time series. For both problems, rather than using simulation models such as the model of the Tennessee Eastman process and the neural mass model of brain networks, we used real datasets. In the Eastman Chemical Company process case, 8 oscillatory process variables are involved, while in the epileptic brain network case, the recorded 50 channels of stereo electroencephalography (sEEG) signals make up our experimental dataset.

### 2.1. Industrial Process Case: Root Cause Analysis of Plant-Wide Oscillations

Oscillation is a common type of plant-wide fault, which could be caused by poorly tuned controllers, valve stiction, or oscillatory disturbances and which propagates to other parts of the plant. In this study, we take the process at Eastman Chemical Company as the industrial process case. The standard dataset of the Eastman process contains the real data of process variables (pv), controller outputs (op), set points (sp), and controller errors. Each variable has 8640 observations (48 h in total) sampled at an interval of 20 s. With power spectra of the variables, the presence of oscillation can be detected at the frequency of about 0.0032 cycles/sample with a period of nearly 2 h. The genuine root cause was identified as LC2.pv. For simplicity, the “.pv” suffix is omitted in the remainder of this article. The oscillation was caused by the valve stiction of LC2, from which it propagated to more related units, resulting in plant-wide oscillations [54]. With the spectral envelope method [55], 8 process variables were recognized as having oscillation faults, which were LC1, FC1, TC1, PC2, FC5, LC2, FC8, and TC2.

### 2.2. Brain Network Case: Localization of Seizure Onset Zones in the Human Brain

Epilepsy is a neurological disorder caused by large-scale abnormal electrical activity in the brain, resulting in temporary brain dysfunction. When an epileptic seizure takes place, abnormal discharges originating from the seizure onset zone (SOZ) will propagate to other regions through oscillatory synchrony between neuronal populations and consequently cause widespread dysfunction in the brain. For patients with drug-resistant epilepsy, surgical resection or laser ablation of the SOZ is a viable treatment. It is crucial to locate the SOZ precisely before surgery. There has been significant research on automatic SOZ localization. One typical approach is to extract features, such as high-frequency oscillations [56], from each channel to differentiate between SOZ channels and non-SOZ ones [57]. Another type of approach is to construct brain effective connectivity networks, based on which the epileptogenic focus can be located [17]. To estimate the effective connectivity between brain regions, some causal detection methods have been commonly employed, including the directed transfer function (DTF) and the partial directed coherence (PDC) [58].

**Figure 1:**
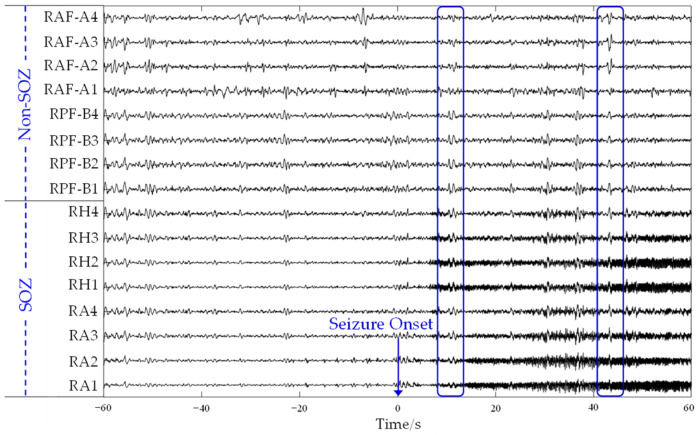
sEEG signals from several channels of a patient in the HUP dataset. The vertical axis labels indicate the channel, and the horizontal axis represents time. A seizure takes place at time 0. There are four channels in each region. Regions RA and RH were identified as SOZs of that patient by the surgical outcome.

In this study, we used the HUP iEEG epilepsy dataset [59]. It contains multichannel iEEG recordings, including electrocorticography (ECoG) signals and sEEG signals, of deidentified patients with drug-resistant epilepsy at the Hospital of the University of Pennsylvania. The iEEG data were collected as part of the surgical treatment, including channels that were clinically diagnosed as the SOZs and annotations indicating which channels were resected or ablated. The multichannel sEEG signals of an epileptic are shown in Figure 1. It can be observed that there is synchronized rhythmic activity (marked by blue boxes) across channels of SOZ regions RA and RH and of non-SOZ region RPF-B. Patient-specific study is necessary. We chose patient HUP116, whose SOZs were verified by the surgical outcomes with an Engel score of IA. The data we used include multichannel sEEG signals from 2 min before the seizure onset to 1 min after the seizure termination. Excluding channels that were marked as bad, 50 channels of 13 brain regions were used in the experiments, where regions RA and RH are the true SOZs of that patient.

## 3. Methods and Results

In Section 3.1, brief descriptions of the mainstream time series causality detection methods are given with a possible taxonomy. The essential definitions of several typical methods are presented here. For more related mathematical descriptions or derivations, interested readers may refer to the Appendix A or the References.

To make a comprehensive comparison, we designed several sets of experiments and implemented 11 mainstream methods separately on each case in a standard way to infer the causal interactions among the time series. Then, based on the detected causalities, the root cause of the fault or failure was analyzed. Finally, with the known root cause of each case, the methods were evaluated within a unified and reasonable framework.

### 3.1. Taxonomy of Causality Detection Methods

**Figure 2:**
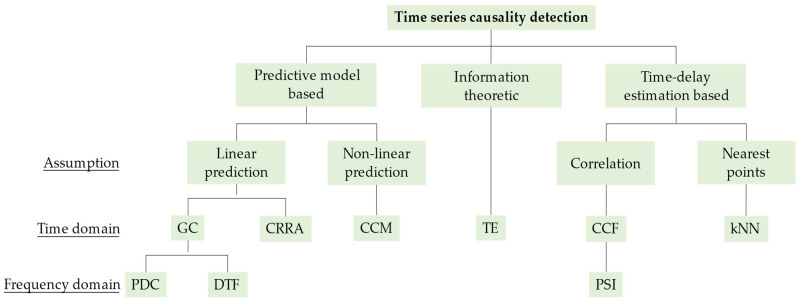
A possible taxonomy of the mainstream causality detection methods. Abbreviations: GC, Granger causality; PDC, partial directed coherence; DTF, directed transfer function; CRRA, causal relationship based on residual analysis; CCM, convergent cross mapping; TE, transfer entropy; CCF, cross-correlation function; PSI, phase slope index; *k*NN, *k* nearest neighbors.

As illustrated in Figure 2, we highlight three branches of time series causality detection methods, i.e., the predictive model-based, information-theoretic, and time delay estimation-based branches, according to the strategy adopted.

#### 3.1.1. Predictive Model-Based Branch

This branch of methods relies on the principle of predictability improvement. Of this branch, the first subdivision is according to the assumption of the predictive model.

A. Linear prediction

This type fits a linear regressive model to predict the effect variable based on the predictors including the cause variable and assesses the causality with criteria describing the goodness of fit of the model.

(1) GC and MVGC

The Granger causality between variables *X* and *Y* can be assessed by comparing the prediction residuals of univariate and bivariate AR models [60]. The univariate AR models for variables *X* and *Y* are described as

(1) $$\begin{array}{c} X\left(t\right)=\sum_{l=1}^{p}\;b_{X,l}X\left(t-l\right)+e_{X\left(Y\right)}\left(t\right) \end{array}$$

(2) $$\begin{array}{c} Y\left(t\right)=\sum_{l=1}^{p}\;\;b_{Y,l}Y\left(t-l\right)+e_{Y\left(X\right)}\left(t\right) \end{array}$$

where $b_{X,l}$ and $b_{Y,l}$ are model coefficients, $e_{X\left(Y\right)}$ and $e_{Y\left(X\right)}$ are prediction errors, and *p* is the model order. By including *Y* and *X* as regression variables in Equations (1) and (2), respectively, the bivariate AR models are expressed as

(3) $$\begin{array}{c} \left[\begin{array}{c} X\left(t\right)\\ Y\left(t\right) \end{array}\right]=\sum_{l=1}^{p}\;\left[\begin{array}{cc} a_{XX,l} & a_{XY,l}\\ a_{YX,l} & a_{YY,l} \end{array}\right]\left[\begin{array}{c} X\left(t-l\right)\\ Y\left(t-l\right) \end{array}\right]+\left[\begin{array}{c} e_{X}\left(t\right)\\ e_{Y}\left(t\right) \end{array}\right] \end{array}$$

where $a_{XX,l}$, $a_{YX,l}$, $a_{YX,l}$, and $a_{YY,l}$ are model coefficients and $e_{X}$ and $e_{Y}$ are prediction errors. For a system with n variables, $X(t)=[X_{1}\left(t\right),X_{2}\left(t\right),\ldots ,X_{n}\left(t\right)]$, Equation (3) can be extended to a MVAR model as shown in Equation (4).

(4) $$\begin{array}{c} \left[\begin{array}{c} X_{1}\left(t\right)\\ X_{2}\left(t\right)\\ \vdots \\ X_{n}\left(t\right) \end{array}\right]=\sum_{l=1}^{p}\;\left[\begin{array}{cccc} a_{11,l} & a_{12,l} & \cdots & a_{1n,l}\\ a_{21,l} & a_{22,l} & \cdots & a_{2n,l}\\ \vdots & \vdots & \vdots & \ldots \\ a_{n1,l} & a_{n2,l} & \cdots & a_{nn,l} \end{array}\right]\left[\begin{array}{c} X_{1}\left(t-l\right)\\ X_{2}\left(t-l\right)\\ \vdots \\ X_{n}\left(t-l\right) \end{array}\right]+\left[\begin{array}{c} e_{1}\left(t\right)\\ e_{2}\left(t\right)\\ \vdots \\ e_{n}\left(t\right) \end{array}\right] \end{array}$$

In the MVAR model described by Equation (4), if variable $X_{j}$ is removed, the model becomes the following:

(5) $$\begin{array}{c} \left[\begin{array}{c} X_{1}\left(t\right)\\ \begin{array}{c} \vdots \\ \begin{array}{c} X_{j-1}\left(t\right)\\ X_{j+1}\left(t\right) \end{array} \end{array}\\ \vdots \\ X_{n}\left(t\right) \end{array}\right]=\sum_{l=1}^{p}\;\left[\begin{array}{cccc} {a^{\prime }}_{11,l} & {a^{\prime }}_{12,l} & \cdots & {a^{\prime }}_{1n,l}\\ \begin{array}{c} \vdots \\ \begin{array}{c} {a^{\prime }}_{\left(j-1\right)1,l}\\ {a^{\prime }}_{\left(j+1\right)1,l} \end{array} \end{array} & \begin{array}{c} \vdots \\ \begin{array}{c} {a^{\prime }}_{\left(j-1\right)2,l}\\ {a^{\prime }}_{\left(j+1\right)2,l} \end{array} \end{array} & \begin{array}{c} \begin{array}{c} \vdots \\ \cdots \end{array}\\ \cdots \end{array} & \begin{array}{c} \vdots \\ \begin{array}{c} {a^{\prime }}_{\left(j-1\right)n,l}\\ {a^{\prime }}_{\left(j+1\right)n,l} \end{array} \end{array}\\ \vdots & \vdots & \vdots & \ldots \\ {a^{\prime }}_{n1,l} & {a^{\prime }}_{n2,l} & \cdots & {a^{\prime }}_{nn,l} \end{array}\right]\left[\begin{array}{c} X_{1}\left(t-l\right)\\ \begin{array}{c} \vdots \\ \begin{array}{c} X_{j-1}\left(t-l\right)\\ X_{j+1}\left(t-l\right) \end{array} \end{array}\\ \vdots \\ X_{n}\left(t-l\right) \end{array}\right]+\left[\begin{array}{c} e_{1\left(j\right)}\left(t\right)\\ \begin{array}{c} \vdots \\ \begin{array}{c} e_{j-1\left(j\right)}\left(t\right)\\ e_{j+1\left(j\right)}\left(t\right) \end{array} \end{array}\\ \vdots \\ e_{n\left(j\right)}\left(t\right) \end{array}\right] \end{array}$$

where $e_{i\left(j\right)}\left(t\right)$ is the prediction error of variable $X_{i}$ when $X_{j}$ is excluded from the regression variables. Equation (4) uses all variables as the regression variables and is called the unrestricted model or full model. Equation (5) is the restricted model. When the MVAR model shown in Equation (4) is employed in the GC inference, the causality inference is referred to as multivariate Granger causality (MVGC).

The difference between the prediction errors of the unrestricted model and the restricted model reflects the difference of prediction capability, which can be used to measure GC between variables. The GC from variable $X_{j}$ to $X_{i}$ is defined as

(6) $$\begin{array}{c} F_{j\to i}=\ln\frac{var\left(e_{i\left(j\right)}\right)}{var\left(e_{i}\right)}. \end{array}$$

$F_{j\to i}$ reflects the difference of residual variance between the restricted model and the full model. The larger the $F_{j\to i}$ is, the stronger the GC from $X_{j}$ to $X_{i}$ will be. Conversely, when $F_{j\to i}$ is close to zero, it indicates the absence of GC.

For the significance test of GC, interested readers may refer to Appendix A.1.

(2) DTF and PDC

Unlike GC that compares the prediction performance of the restricted model and the full model, the directed transfer function (DTF) and partial directed coherence (PDC) measure the causal effect based on the coefficients in the full model. The MVAR model in Equation (4) can be rewritten in the form of

(7) $$\begin{array}{c} X\left(t\right)=\sum_{l=1}^{p}A\left(l\right)X\left(t-l\right)+E\left(t\right) \end{array}$$

where $X\left(t\right)=[x_{1}\left(t\right)x_{2}\left(t\right)\ldots x_{n}(t)]$ is the *n*-dimensional signal at time t, $E\left(t\right)=[e_{1}\left(t\right)e_{2}\left(t\right)\ldots e_{n}(t)]$ is the prediction error vector at time t, and $A\left(l\right)$ is the n×n coefficient matrix with delay l, with matrix elements $A_{ij}\left(l\right)$ representing the influence of $x_{j}(t-l)$ on $x_{i}(t)$. In Equation (7), by moving $X\left(t\right)$ and $\sum_{l=1}^{p}A\left(l\right)X\left(t-l\right)$ to the same side and performing a Fourier transform on both sides, we have

(8) $$\begin{array}{c} E\left(f\right)=A\left(f\right)X\left(f\right) \end{array}$$

where

(9) $$\begin{array}{c} A\left(f\right)=-\sum_{l=0}^{p}A\left(l\right)e^{-2\pi i\frac{f}{f_{s}}l} \end{array}$$

where $f_{s}$ is the sampling frequency of the time series, $A\left(0\right)=-I_{n\times n}$ (the n×n identity matrix), and $E\left(f\right)$, $X\left(f\right)$, and $A\left(f\right)$ are the results of the Fourier transform of the error sequence, original time series, and model coefficients, respectively. Let $H(f)=A^{-1}\left(f\right)$, then Equation (8) can be rewritten in the form of Equation (10). Here, H(f) is defined as the transfer function of the system. H(f) is an n×n matrix with element $H_{ij}(f)$ representing the influence of variable $x_{j}$ on $x_{i}$ at frequency f.

(10) $$\begin{array}{c} X\left(f\right)=H\left(f\right)E\left(f\right) \end{array}$$

The DTF from variable $X_{j}$ to variable $X_{i}$ at frequency f is defined as the division of the elements $H_{ij}\left(f\right)$ by the squared sum of all the elements of the relevant row and is calculated using

(11) $$\begin{array}{c} \gamma_{ij}\left(f\right)=\frac{H_{ij}\left(f\right)}{\sqrt{\sum_{k=1}^{n}{\left|H_{ik}\left(f\right)\right|}^{2}}}. \end{array}$$

For $X_{i}$, the normalization condition takes the form of

(12) $$\begin{array}{c} \sum_{k=1}^{n}\gamma_{ik}^{2}\left(f\right)=1. \end{array}$$

Therefore, DTF measures the influence on variable $X_{i}$ at each frequency from all variables in the system [61].

According to Equation (8), the PDC from variable $X_{j}$ to variable $X_{i}$ is given by

(13) $$\begin{array}{c} \pi_{ij}\left(f\right)=\frac{A_{ij}\left(f\right)}{\sqrt{\sum_{k=1}^{n}{\left|A_{kj}\left(f\right)\right|}^{2}}} \end{array}$$

For $X_{j}$, the normalization condition takes the form of

(14) $$\begin{array}{c} \sum_{k=1}^{n}\pi_{kj}^{2}\left(f\right)=1. \end{array}$$

Therefore, the PDC measures the influence from variable $X_{j}$ at each frequency component on all variables in the system [62,63].

For improvements of the DTF and PDC, interested readers may refer to Appendix A.2.

B. Nonlinear prediction

A typical example of a nonlinear prediction model-based method is convergent cross mapping (CCM). It is noteworthy that despite most time series-based causality analysis methods being implementations of the maxim that causes precede and predict their effects, the idea of CCM is that if causation exists, the effect variable must contain information about the cause variable.

(1) CCM

At time t, the embedding vectors of the shadow manifolds $M_{X}$ and $M_{Y}$ of variables *X* and *Y* are denoted as

(15) $$\begin{array}{c} m_{X}\left(t\right)=\left[x_{t},x_{t-\tau },\ldots ,x_{t-\left(l-1\right)\tau }\right] \end{array}$$

(16) $$\begin{array}{c} m_{Y}\left(t\right)=\left[y_{t},y_{t-\tau },\ldots ,y_{t-\left(l-1\right)\tau }\right] \end{array}$$

CCM predicts the ${\hat{m}}_{X}$ of $M_{X}$ at a new time t using the points in $M_{X}$ that are mapped to the nearest neighbors of $m_{Y}\left(t\right)$ in $M_{Y}$ [64]. The K nearest neighbors of $m_{Y}\left(t\right)$ in $M_{Y}$ are denoted as $m_{Y}\left(t_{i}\right)$ for i=1,2,…,K, with $m_{Y}\left(t_{1}\right)$ being the nearest neighbor. ${\hat{m}}_{X}\left(t\right)$ is estimated by Equation (25), which uses the mapping of the nearest neighbors $m_{Y}\left(t_{i}\right)$ in $M_{Y}$, i.e., $m_{X}\left(t_{i}\right)$.

(17) $$\begin{array}{c} \left.{\hat{m}}_{X}\left(t\right)\right|M_{Y}=\sum_{i=1}^{K}w_{i}m_{X}\left(t_{i}\right) \end{array}$$

where *K* is the number of neighbors used for prediction, and

(18) $$\begin{array}{c} w_{i}=\frac{u_{i}}{\sum_{j}u_{j}} \end{array}$$

(19) $$\begin{array}{c} u_{i}=\exp\left(-\frac{d\left[m_{Y}\left(t_{i}\right),m_{Y}\left(t\right)\right]}{d\left[m_{Y}\left(t_{1}\right),m_{Y}\left(t\right)\right]}\right) \end{array}$$

where $d\left[m_{Y}\left(t_{i}\right),m_{Y}\left(t\right)\right]$ is the Euclidean distance between the vectors. At a given time, the cross-mapping prediction effect is represented by the correlation coefficient of the first element, denoted as X, of the true embedding vector $m_{X}$ and the first element, denoted as X and $\left.\hat{X}\right|M_{Y}$, of the predicted vector $\left.{\hat{m}}_{X}\right|M_{Y}$.

(20) $$\begin{array}{c} \rho_{X\to Y}=\underset{L\to \infty }{lim}\frac{cov\left(X,\left.\hat{X}\right|M_{Y}\right)}{\sigma_{X}\sigma_{\left.\hat{X}\right|M_{Y}}} \end{array}$$

When *L* is sufficiently large, $\rho_{X\to Y}$ represents the causal effect from *X* to *Y*. The better the estimation of the cross mapping of *Y* to *X* is, the stronger the causal effect from *X* to *Y* will be.

#### 3.1.2. Information-Theoretic Branch

A kind of generalized information-theoretic method is transfer entropy (TE), along with its extensions, which detect delayed interactions between time series [65,66]. They are able to detect nonlinear forms of interaction, which may be invisible to linear methods like GC. As it is model-free, a priori assumption is not required on connectivity patterns.

(1) TE

The transfer entropy from variable *X* to *Y* is defined as

(21) $$\begin{array}{c} T_{X\to Y}=H\left(Y_{t+h}|Y_{t}\right)-H\left(Y_{t+h}|X_{t},Y_{t}\right) \end{array}$$

where h is the prediction horizon. $H\left(Y_{t+h}|Y_{t}\right)$ and $H\left(Y_{t+h}|X_{t},Y_{t}\right)$ are conditional entropies. Equation (21) is the essential definition. Interested readers may refer to Appendix A.3 for the calculation of TE as well as an extension of the TE method that is able to rule out parts of indirect causal relations.

#### 3.1.3. Time Delay Estimation-Based Branch

Methods of this branch estimate the time delay between a pair of variables. The interactions exert their largest influence at a specific time delay.

(1) CCF

The cross-correlation function (CCF) first calculates the linear correlation between two variables at each lag [3]. For variables *X* and *Y*, we have

(22) $$\begin{array}{c} \rho_{XY}\left(\tau \right)=\frac{E\left[\left(x_{i}-\mu_{X}\right)\left(y_{i+\tau }-\mu_{Y}\right)\right]}{\sigma_{X}\sigma_{Y}} \end{array}$$

where μ and σ are the mean and standard deviation of the variable. When the absolute value of the correlation between variables *X* and *Y* is maximized, the lag is

(23) $$\begin{array}{c} {\hat{\tau }}_{XY}=\underset{\tau_{\min}\leq \tau \leq \tau_{\max}}{\mathrm{argmax}}\left|\rho_{XY}\left(\tau \right)\right| \end{array}$$

where $\tau_{min}$ and $\tau_{max}$ are the minimum and maximum lags considered, respectively. ${\hat{\tau }}_{XY}>0$ indicates that *X* causes Y. The causal effect between X and Y can be quantified by $\rho_{XY}\left({\hat{\tau }}_{XY}\right)$. With the CCF, not only is the causal effect $\rho_{XY}\left({\hat{\tau }}_{XY}\right)$ between two variables measured but also the time lag at maximal correlation ${\hat{\tau }}_{XY}$ can be retained.

(2) PSI

The phase slope index (PSI) is a frequency-domain method that in some way quantifies the consistency across observations of the phase difference between the oscillatory components in the signals [67]. It is defined as

(24) $$\begin{array}{c} {\tilde{\psi }}_{XY}=Im\left(\sum_{f\in F}C_{XY}^{*}(f)C_{XY}(f+\delta f)\right) \end{array}$$

where Im(·) represents the imaginary part, * represents complex conjugation, F is the selected frequency set, δf is the frequency resolution, and $C_{XY}\left(f\right)$ is the complex coherence, which is defined as

(25) $$\begin{array}{c} C_{XY}\left(f\right)=\frac{S_{XY}\left(f\right)}{\sqrt{S_{XX}\left(f\right)S_{YY}\left(f\right)}} \end{array}$$

where $S_{XY}\left(f\right)$ is the cross-spectral density function of variables X and Y.

In this study, a total of 11 causality detection methods that are popular or up-to-date were investigated, including TE, GC, CCM, DTF, PDC, and PSI, as well as a few of their extensions.

As for the experimental environment, in this study we used a PC running Linux version 5.3.0-46-generic with an Inter(R) Core (TM) i9-10900K CPU and 64 GB of RAM. The programs were developed and executed with Matlab R2023a.

### 3.2. RCA of Plant-Wide Oscillations

On the problem of analyzing the root cause of plant-wide oscillations of the Eastman process, we designed the following three sets of experiments, where the effects of applying different causality detection methods can be observed and compared.

#### 3.2.1. Time-Domain Methods

First, we implemented five time-domain causal inference methods, including the bivariate GC, TE, and CCM, as well as the multivariate methods MVGC and direct TE (DTE). Interested readers may refer to Appendix A.4 for descriptions of DTE. Then, based on the causal relations detected by each method, a causal graph was constructed for graphically modeling and reasoning about the fault spreading behaviors in the form of causal dependencies across the oscillatory variables. The parameter settings and how some of them were determined for each method are outlined in Table 2.

To identify whether a causal relation is significant or not, different from GC and MVGC that use a null hypothesis test, TE and DTE employ a Monte Carlo simulation. With that method, we generated 3000 couples of surrogate data for the original time series. Then, TE or DTE metrics were calculated between each couple of surrogate data. Their mean plus three times the standard deviation was set as the threshold. If the TE or DTE metrics calculated from the original time series exceeded the threshold, it indicated a significant causal relation between the pair of the original time series.

With respect to CCM, its results and discussions are presented in Section 3.4, where the similarities and differences of CCM applied in both cases can be compared more clearly.

**Figure 3:**
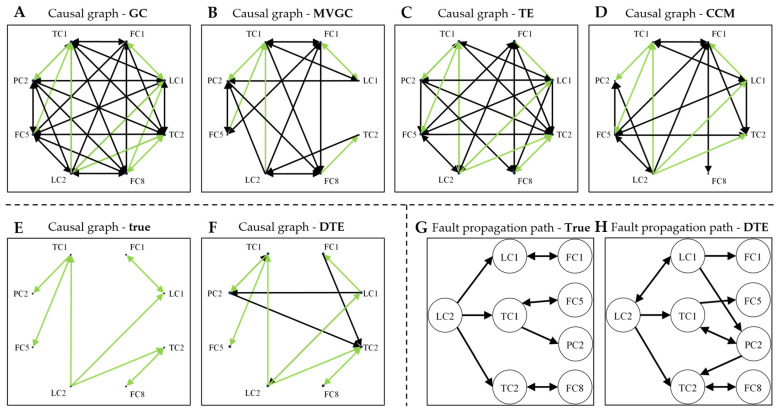
Causal graphs and fault propagation paths. (**A**–**D**,**F**): estimated from different causal detection methods; (**E**): the true causal relations among oscillatory process variables; (**G**,**H**): the true fault propagation path and the path obtained with DTE. The arrows denote a directional connection between two variables. Arrows that fit the true causal relations are marked in green.

Based on the detected causalities between each pair of oscillatory variables, a causal graph resulting from each causality detection method can be constructed, as depicted in Figure 3A–D,F. The true causal relations among those variables that were obtained by use of the process knowledge are depicted in Figure 3E.

As can be seen from the figure, both GC and TE found too many false-positive causal relations. By MVGC, 6 causal relations were identified correctly. But still, 17 false positives were yielded. In contrast, DTE identified a total of 12 causal relations, 8 of which fit the true situation. Figure 3G,H show the true propagation path and that obtained with DTE. They indicate that the propagation path resulting from DTE was close to the true situation.

#### 3.2.2. Frequency-Domain Methods

**Figure 4:**
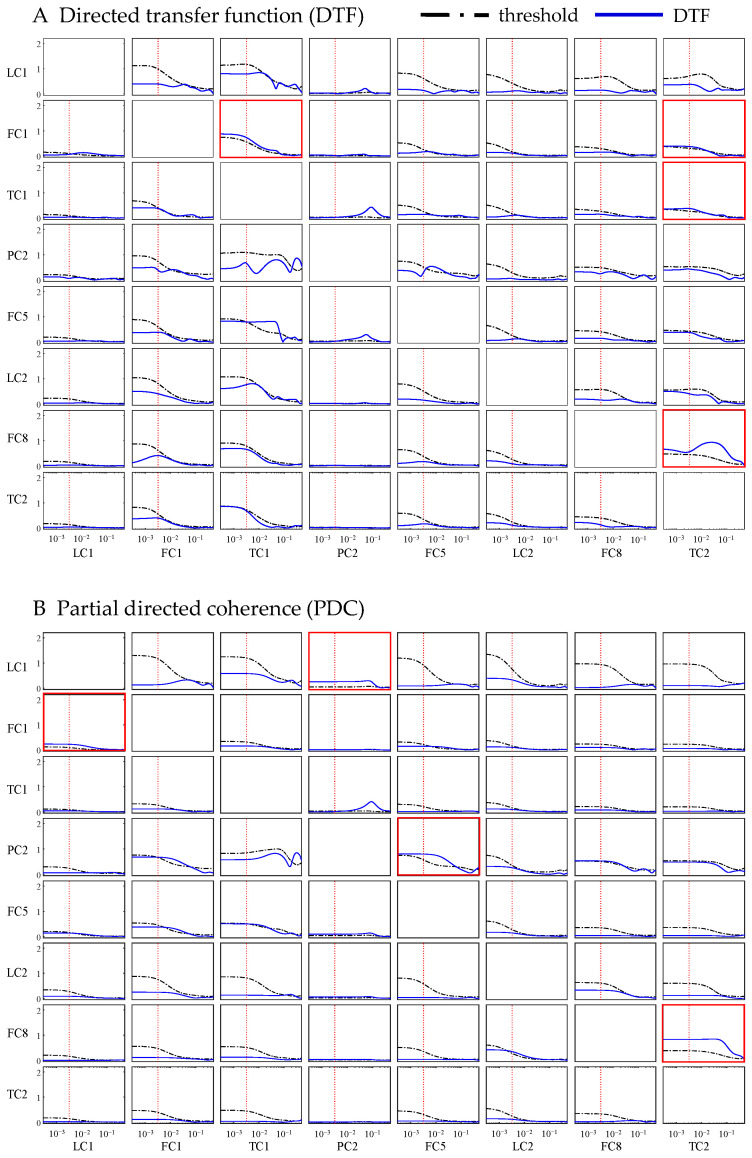
Causalities detected by DTF and PDC. Each subgraph shows the causality from the row variable to the column variable. In each subgraph, the vertical axis represents causality and the horizontal represents frequency. The blue curve represents the calculated causality varying with frequency, while the black dotted curve is the significance threshold determined with the Monte Carlo simulation. The red vertical dashed line marks the oscillation frequency of the process. Subgraphs where the causal effects are significant at the oscillation frequency are marked with red boxes.

Several methods that quantify causal effects from the perspective of the frequency domain were implemented, including the DTF and the PDC. The parameter settings and how some of them were determined are outlined in Table 3. The resulting curves of causality of each pair of variables detected by the DTF and PDC, along with the corresponding significance threshold curves, are shown in Figure 4A,B, respectively. Subgraphs where the causal effects are significant at the oscillation frequency are marked with red boxes. It can be seen that with either the DTF or the PDC, only a few causations were significant at the oscillation frequency. These two frequency-domain methods failed to yield promising results.

#### 3.2.3. Improved Frequency-Domain Methods

The spectrum-weighted DTF (swDTF) and the spectrum-weighted PDC (swPDC) are improved frequency-domain methods that weight causality at different frequencies by the power spectral density; thus, the frequency components with higher spectral energy could be counted more. With the detected directed relations between each pair of variables, a causal network could be constructed for graphical reasoning. A node measure, the causal information outflow, is used, as defined below:

$$C{ausal\_information\_flow}_{X_{i}}=\sum_{j=1,\;j\neq i}^{n}{Causality}_{X_{i}\to X_{j}}$$

**Figure 5:**
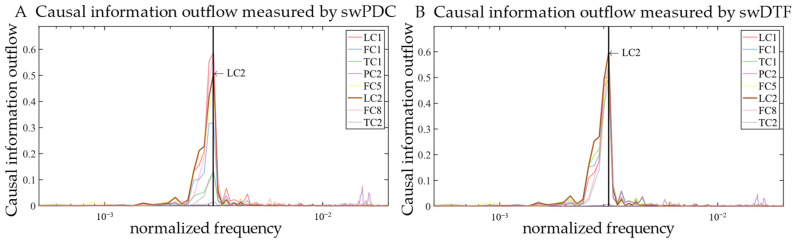
Curves of causal information outflow varying with frequency of each oscillatory variable measured by swDTF and swPDC, respectively. The black vertical line indicates the common oscillation frequency.

The curve of causal information outflow of each variable with frequency, measured by the swDTF and swPDC, are shown in Figure 5, respectively. Considering that all the variables oscillate at a common frequency, we marked that frequency on the information outflow curve with a back vertical line. It is observed that with the swDTF, LC2 had the highest information outflow at the common oscillation frequency. Since LC2 was the true root cause, this observation suggests that the swDTF is useful for RCA in this industrial case. However, swPDC could not yield such conclusions.

### 3.3. Localization of SOZs in the Human Brain

**Figure 6:**
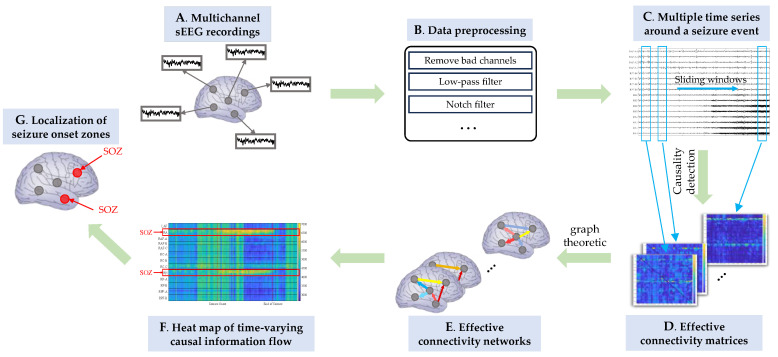
The schematic diagram of SOZ localization with brain effective connectivity network.

In the brain network case, according to characteristics of the SOZ localization problem, we designed experiments that are different from those in the industrial process case. The schematic diagram is shown in Figure 6. First, the connectivity patterns between brain regions or neuronal groups are transient and unstable, which is different from the relatively stable relations between variables in industrial processes. To detect the transient causal interactions, we calculated the effective connectivity with sliding time windows. Second, quantifying the driving force of each related brain region during a seizure is helpful to locate the SOZs, whose driving forces would be higher. For each time window, when the brain effective connectivity network is constructed based on the estimated causal relations between channels, causal information outflow of each channel can be calculated, which represents the driving force of that channel. The varying information outflow of each channel over time reflects the dynamic trends of the connection pattern in the brain network. In this way, neurobiological inferences can be made.

**Figure 7:**
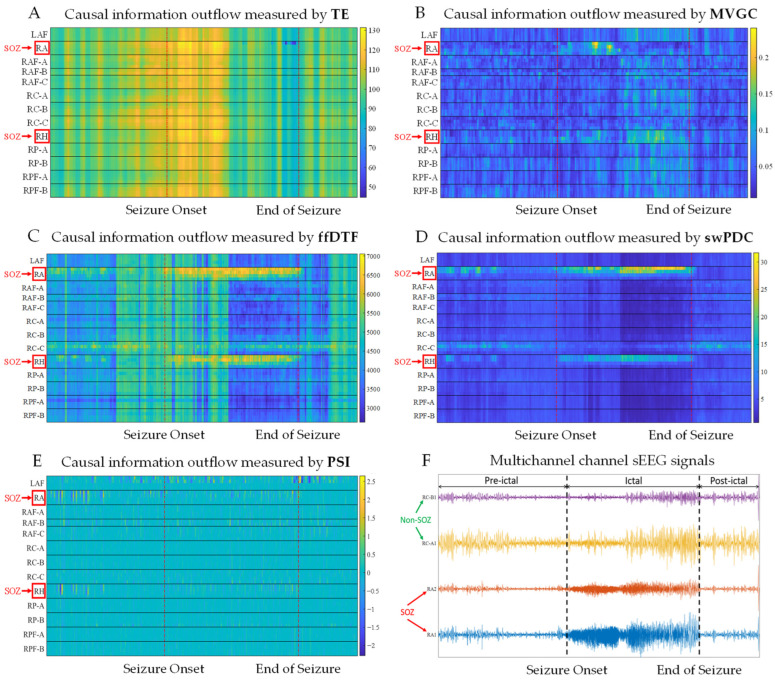
Heatmaps of causal information outflow of each channel varying over time, which resulted from different causal inference methods. In each subgraph, the horizontal axis represents time and the vertical labels indicate the brain regions to which the channel belongs. The amount of causal information outflow is represented by color.

In the brain network case, we implemented the TE, MVGC, full-frequency DTF (ffDTF), spectrum-weighted DTF (swDTF), spectrum-weighted PDC (swPDC), and PSI methods separately to estimate the effective connectivity among the 50 channels. The results needed to be presented in a unified way to facilitate evaluation and comparison. For that reason, with respect to each of ffDTF, swDTF, and swPDC, the causalities at all frequencies within the considered frequency band were summed to represent an overall causal effect. Figure 7 shows several representative heatmaps of causal information outflow of each channel varying over time, which resulted from parts of the methods we implemented. The signal of each channel was low-pass-filtered at 1~60 Hz. We used a sliding window with a width of 5 s and a step of 0.2 s. Therefore, each data segment contains 2500 data points. Table 4 shows the parameter settings and how some of them were determined for each method.

As shown in the figure, with the ffDTF and swPDC, the channels of brain regions RA and RH had much higher causal information outflow than the others. These two brain regions were correctly identified as SOZs. Such findings fit the true situation well. As for MVGC, it resulted in only a slight differentiation between the RA channels and the others. With respect to the other methods, i.e., TE, swDTF, and PSI, neither RA nor RH could be identified.

**Figure 8:**
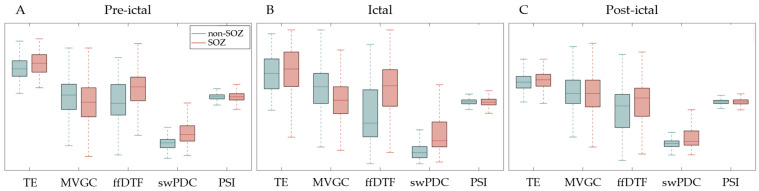
Boxplots of distributions of causal information outflow of SOZ channels and non-SOZ channels that resulted from different methods. Green and red boxes represent non-SOZ and SOZ, respectively.

To compare the ability of different methods to distinguish SOZ channels from non-SOZ channels, we made boxplots of the distributions of information outflow that resulted from those methods separately, as shown in Figure 8. Green and red boxes represent non-SOZ and SOZ channels, respectively. In the statistics, a sample refers to the information outflow within a given time window of a single channel. Samples of preictal, ictal, and postictal periods from SOZ channels and non-SOZ channels were counted separately.

It can be observed that when using the ffDTF and swPDC, the causal information outflows of SOZ channels are generally greater than those of non-SOZ channels. Also, this difference remains consistent in both the preictal and ictal periods. It indicates that both methods effectively located the SOZ in either the interictal or the ictal period and outperformed the other methods. During the postictal period, the difference of information outflows between SOZ and non-SOZ channels becomes smaller. It aligns with the clinical observation that the driving force of the SOZ tends to decline after the end of a seizure.

### 3.4. CCM in Both Cases

In the following, the application of CCM to both cases is presented. In CCM analysis, the skill of cross-map estimates is represented by a correlation coefficient that is defined in Equation (20), which we rewrite as follows:

$$\rho_{X\to Y}=\underset{L\to \infty }{lim}\frac{cov\left(X,\left.\hat{X}\right|M_{Y}\right)}{\sigma_{X}\sigma_{\left.\hat{X}\right|M_{Y}}}$$

where $\rho_{X\to Y}$ is the correlation coefficient between the cross-map estimate $\left.\hat{X}\right|M_{Y}$ and the true value X. For instance, in the cross mapping of *X* on the manifold of *Y*, if ρ converges with the increasing length of the time series used in the calculation, it indicates the presence of a causal effect of *X* on *Y*, and the effect is quantified by the convergence value of ρ. The closer *ρ* is to +1 or −1, the stronger the causal effect will be, while ρ=0 indicates no causal relation.

**Figure 9:**
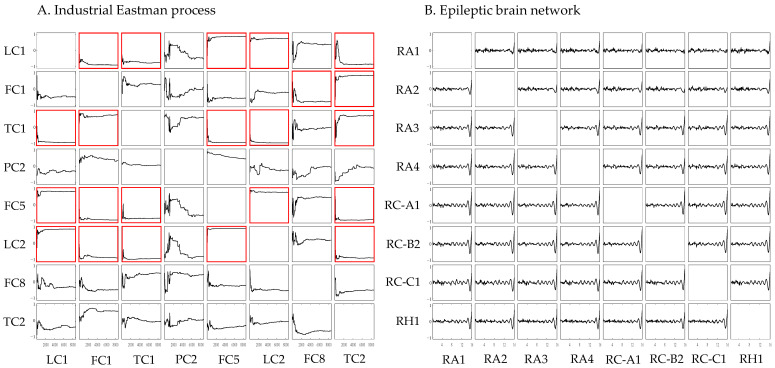
Convergence of the correlation coefficient *ρ* in computing the CCM between each pair of variables of the Eastman process (**A**) and between several of the total 50 channels of the epileptic brain (**B**) with the increasing length of the time series used in the calculation. In each subgraph, the horizontal axis represents the time series length, and the vertical axis represents the correlation coefficient *ρ* between the true value X and the value $\left.\hat{X}\right|M_{Y}$ estimated by cross mapping, which indicates the estimation ability of cross mapping the *row* variable using the manifold of the *column* variable. Subgraphs where ρ converges to a value with absolute value greater than 0.75 are marked with red boxes.

Figure 9 shows the convergence of *ρ* in the industrial Eastman process case (Figure 9A) and in the epileptic brain case (Figure 9B), respectively. Each figure shows the evolving correlation coefficient (ρ) with the increasing length of the time series in CCM calculation for each pair of variables in the Eastman process (A) or each pair of channels in the brain (B).

In the brain network case, as can be seen from the right figure, the ability of cross-map estimates, characterized by *ρ*, between every pair of channels does not converge. This indicates that CCM turns out to be inapplicable and thus is unable to give a meaingful result.

In contrast, in the Eastman process, the convergence of ρ seems much better. For instance, as shown in the left figure, in the cross mapping of LC2 on the manifold of LC1, ρ converges to a value close to 1, indicating a strong causal effect of LC2 on LC1. The strength of the causal relation is quantified by the convergence value of ρ. In the figure, subgraphs where *ρ* converges to a value with an absolute value greater than 0.75, which can be viewed as indicating significant connectivity, are marked with red boxes. Table 5 shows the convergence value of *ρ* for each pair of variables. Each element represents the causal relation from the row variable to the column variable. As a result, the binary causal graph determined with CCM is shown in Figure 3D. Out of the 10 true causal relations (highlighted in blue in Table 5), 6 are correctly identified. However, CCM also yields some false-positive causal relations.

## 4. Discussion

### 4.1. RCA of Plant-Wide Oscillations

In the RCA of plant-wide oscillations in the Eastman process, it is obvious that either GC or TE finds too many causal relations. This is because indirect causal relations are also detected. A bivariate causality analysis such as GC and TE is based on only the pair of candidates. It compares the univariate prediction of *Y* to the bivariate prediction of *Y* that is based on past values of both *X* and *Y*. Consequently, the detected causation may be a combination of the direct influence between X and Y and the indirect influence through other variables, such as common causes or intermediate variables.

The above issue can be much better addressed by MVGC and DTE, which are capable of excluding some of the indirect, redundant causal relations. Multivariate methods like MVGC investigates the impact of removing a given variable *X_j_* on the multivariate prediction of *X_i_* based on all the *n* predictors *X*_1_, …, *X_n_*. In this way, some redundant causal relations can be ruled out. In terms of DTE, by choosing the common cause variables or intermediate variables from the causal relations that have been detected with TE as the conditional variable, given which the DTE measures the direct effect of the cause variable on the effect variable, it rules out indirect causal relations.

DTE closely reflects the true causal relations compared to MVGC. With DTE, the oscillation was identified as originating mainly from LC1 and LC2. This is close to the true situation. In fact, in order to identify the root cause more accurately, process knowledge such as piping and instrumentation diagrams (P&IDs) are valuable to be used in combination with measured data as a complimentary source.

With respect to CCM, it yields some false-positive causal relations. This is in part due to the strong coupling across the variables caused by the oscillation at a common frequency.

To sum up, in the industrial Eastman process case, of all the implemented methods, the time-domain multivariate methods MVGC and DTE were the most reliable. Of the two methods, DTE, which is able to detect nonlinear causal relations, is more reliable than the linear model-based MVGC. As for the frequency-domain methods, they were not as appropriate in this case, although the swDTF provided some valuable information for reference.

### 4.2. Localization of SOZs in the Human Brain

i. The GC and TE methods, which are widely used for identifying the root cause of fault in industrial processes, work poorly on brain networks. On one hand, bivariate causality detection methods (TE, GC, and PSI) cannot rule out indirect, redundant causal relations. As a consequence, the causal information outflow that results from any of them shows similar trends across SOZ and non-SOZ channels, making it difficult to identify the SOZ. On the other hand, to address the requirement for stationarity of the time series used in the causal inference in this case, we used sliding windows whose width was limited. However, both TE and GC have a high requirement for the number of samples.
ii. Multivariate methods (ffDTF, swPDC, and MVGC) are more effective. Of those methods, the ffDTF and swPDC, which detect causal effects in the frequency domain, produced the most promising results.
iii. As for PSI, it may be inapplicable in this case. Bidirectional interactions are the dominant interaction scenario in the majority of cortico–cortical connections. In such situations, the interpretation of the phase difference spectrum (and consequently PSI) as well as the CCF becomes complicated and may fail at correctly describing the directionality. See Witham et al. (2011) and Vinck et al. (2015) for further discussion [68,69].
iv. In brain networks, CCM does not converge and consequently fails to discover causations. A possible reason for the nonconvergence is that CCM assumes that the dynamic system is described by a nonlinear model and has a specific trajectory. However, the dynamics of the brain connectivity pattern changes with time.

### 4.3. Summary

To summarize the above case studies, several conclusions can be drawn as follows: (i) It is mainly the linear predictive model-based methods as well as the information-theoretic methods that were shown to be the most effective. (ii) However, there was no common promising method across both cases. (iii) In the Eastman process, it was two time-domain methods, MVGC and DTE, that outperformed the others, partly due to their ability to rule out indirect causations. (iv) In contrast, in the brain networks, it was extensions of the two frequency-domain methods based on multivariable linear predictive models, i.e., ffDTF and swPDC, that contributed to finding the SOZs accurately. (v) As for CCM and PSI, both of them failed to identify the root cause(s) in either case. (vi) Most methods were designed for relatively stable interaction patterns. As a consequence, when it comes to brain network, the causal connectivity is more challenging to infer as it changes over time.

A quick reference is provided in Table 6 that characterizes the main causality detection methods.

### 4.4. Discussions of Interpretative Pitfall

When it comes to interpreting the causality estimated with data, an issue that warrants caution is whether the estimate reflects a genuine interaction between variables. There are various situations that may lead to nonzero estimates of causal effects in the absence of true interactions. Some of the situations are as follows. The main cause is the fact that in the measured data, the signals are always to some extent mixtures of signals of interest and signals of no interest, which are called “noise”. Thus, when the SNR is different, different interactions will be detected, as will be encountered in brain connectivity network estimation based on different types of neuroimaging recordings. Another cause of spurious estimates is related to interpretation of the observed connectivity pattern. In fact, it is impossible to state whether an estimated relation is direct or not or whether the relation is mediated through an unmeasured variable. In general, if not all variables of the process are taken into calculation, it is impossible to discern whether an estimated relation is direct or not.

To rule out the spurious causality, on one hand, intervention experiments can be conducted as supplementary means if conditions permit. By randomized controlled trials, we can force the value of one variable to change and observe its effect on the other variable. On the other hand, the choice of method should always be guided by the underlying assumptions or hypothesis. Take the brain network issue as an example: if we believe in the hypothesis that nonlinear forms of coupling govern the neuronal interactions, nonlinear methods such as PSI will characterize this kind of relation. PSI focuses on cross-frequency interactions, that is, one nonlinear form of interaction where the amplitude or phase at one frequency interacts with the amplitude or phase at another frequency. If we are interested in the oscillatory phase coupling between neuronal populations, linear approaches are sufficient to characterize a large number of oscillatory relations. Thus, methods such as the phase difference spectrum slope would be needed.

### 4.5. Future Directions

There still remain a lot of open problems. Here, we highlight one of them, i.e., causality detection in a dynamic environment. Existing work mainly focuses on static observations. In practice, data are often continuously collected from a dynamic environment. As we discussed in the brain network case, causal relations can greatly change over time. In an industrial process, the stationary assumption does not necessarily hold due to noises or multimodality in the processes. Thus, developing methods to overcome stationarity restriction is necessary. However, an important requirement in the mainstream causality detection methods is the stationarity of time series. To address this problem, we suggest that machine learning can be considered to bring powerful algorithms. For example, to model dynamic observational data, based on existing work in [44,45], further improvements can be made by employing a neural network to act as a parameterized nonlinear prediction model for a nonlinear form of Kalman filter algorithm, thus to form a time-varying MVAR, which is the basis of MVGC, DTF, and PDC.

## Figures and Tables

**Table 1:** Distinctions between the two systems that we selected for this study from distinct domains.

	Propagation Networks of Faults in Industrial Processes	Effective Connectivity Networks of Human Brains
Stationarity of time series	Nearly stationary or nonstationary [49]	Nonstationary [50]
Stability of connection pattern	Relatively stable in a given state	Transient and unstable
System dynamics	Linear or nonlinear	Nonlinear and with chaos behaviors [40,51]
Network characteristic	Regular or complex [52]	Complex [53]
A priori knowledge	Piping and instrumentation diagrams (P&IDs), etc. [25]	Lacking

**Table 2:** Parameter settings and how some of them were determined for each method.

Method	Parameter Settings
GC MVGC	▪ AR/MVAR model order: 8, determined by the Bayesian information criterion (BIC). ▪ AR/MVAR model parameters estimation: the least squares method. ▪ Identification of significant causality: null hypothesis testing with confidence level of 0.001.
TE DTE	▪ Prediction horizon: 1, i.e., 20 s. ▪ Identification of significant causality: Monte Carlo simulation. ▪ Number of couples of surrogate data: 3000. ▪ Significance threshold: mean + 3SD.
CCM	▪ Dimension of the embedding vector: 18. ▪ Delay of the embedding vector: 1. ▪ Number of nearest neighbors used for cross-map estimation: 8.

**Table 3:** Parameter settings and how some of them were determined in the DTF and the PDC.

	Parameter Settings in DTF and PDC
MVAR model	▪ Criterion to determine the order: BIC. ▪ Determined order: 8. ▪ Model parameters estimation: the least squares method.
Significance determination	▪ Method: Monte Carlo simulation. ▪ Number of couples of surrogate data: 3000. ▪ Threshold: mean + 2SD.

**Table 4:** Parameter settings and how some of them were determined for different methods.

Method	Parameter Settings	
TE	▪ Prediction horizon: 1, i.e., 0.002 s.	
MVGC	▪ Criterion to determine MVGC model order: BIC, for each time window separately.	
ffDTF swDTF swPDC	▪ Criterion to determine MVGC model order: BIC, for each time window separately. ▪ Frequency band counted: 1–30 Hz.	
PSI	▪ Frequency band counted: 1–30 Hz.	

**Table 5:** Causality that resulted from CCM in the Eastman process. Each element represents the causal relation from the row variable to the column variable. Nonconvergent correlation coefficients are indicated by “\”. *True* causal relations are highlighted in *blue*.

	LC1	FC1	TC1	PC2	FC5	LC2	FC8	TC2
**LC1**		−0.90	−0.75	\	0.87	0.75	0.37	−0.84
**FC1**	−0.48		0.28	\	−0.55	−0.19	−0.77	0.84
**TC1**	−0.91	0.81		0.66	−0.91	−0.93	0.01	0.78
**PC2**	−0.30	0.38	0.10		\	\	\	\
**FC5**	0.83	−0.91	−0.79	\		0.80	0.49	−0.89
**LC2**	0.93	−0.85	−0.90	\	0.96		0.24	−0.86
**FC8**	−0.31	−0.45	0.58	0.51	−0.19	−0.49		−0.46
**TC2**	\	0.64	\	\	\	−0.01	−0.59	

**Table 6:** Overview of distinctions among main causality detection methods.

	TE	GC	k-NN	CRRA	CCM	CCF	DTF	PDC	PSI
Time domain	√	√	√	√	√	√			
Frequency domain							√	√	√
Linear relation	√	√	√	√		√	√	√	√
Nonlinear relation	√				√				
Parameterized		√		√			√	√	
Nonparametric	√		√		√	√			√
Bivariate	√	√	√	√	√	√	√	√	√
Multivariate		√		√			√	√	√
Number of samples required	high	high	low	high	medium	low	high	high	low
Computational load	high	low	medium	low	medium	low	medium	medium	low
Insensitive to noise				√			√	√	√
Direct causality	√ ^a^	√ ^b^		√				√	
Number of a priori parameters ^c^	$4(h,l_{1},l_{2},\tau$)	1(p)	3(K,l,τ)	1(p)	3(K,l,τ)	0	1(p)	1(p)	0

“√” denotes yes. a: Original TE cannot detect, while its extensive form DTE can. b: Bivariate GC cannot detect, but its multivariate form MVGC can. c: The parameters are listed in parentheses after the numbers.

**Figure A1:**
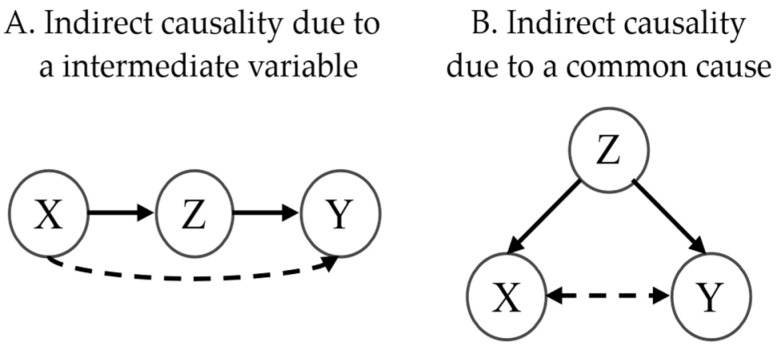
Illustration of indirect causal relations: solid arrows represent actual causal relationships, while dashed arrows represent an indirect causal relation.

## Data Availability

The original data presented in the study are openly available in the PID benchmark database at https://sacac.org.za/resources/ (accessed on 12 November 2023) and the HUP iEEG Epilepsy Dataset at https://openneuro.org/datasets/ds004100/versions/1.1.3 (accessed on 14 January 2024).

## Appendix A. Supplementary Introduction to Time Series Causality Detection Methods

**Table A1:** Notations.

**Parameter/Variable**	**Meaning**
X	“Cause” variable in bivariate causal relations
Y	“Effect” variable in bivariate causal relations
$X_{j}$	“Cause” variable in multivariate causal relations
$X_{i}$	“Effect” variable in multivariate causal relations
p	Order of AR or MVAR model
$m_{X}\left(t\right)$	Shadow manifold of variable X at time t: $m_{X}\left(t\right)=\left[x_{t},x_{t-\tau },\ldots ,x_{t-\left(l-1\right)\tau }\right]$
${\vec{x}}_{t}$	Embedding vector of variable X at time t: ${\vec{x}}_{t}=[x_{t},x_{t-\tau },\ldots ,x_{t-\left(l-1\right)\tau }]$
l	Dimension of the shadow manifold or embedding vector
τ	Time delay of the shadow manifold or embedding vector
K	Number of neighbors used in prediction of CCM or *k*-NN

### Appendix A.1. Significance Test of GC

To determine whether the GC from $X_{j}$ to $X_{i}$ is significant, the following F test can be applied:

(A1) $$\begin{array}{c} F_{statistic}=\frac{\frac{\left({RSS}_{0}-{RSS}_{1}\right)}{p1-p2}}{\frac{{RSS}_{1}}{m-p2}}\sim F_{p1-p2,m-p2} \end{array}$$

where ${RSS}_{0}=\sum_{t}e_{i\left(j\right)}^{2}(t)$, ${RSS}_{1}=\sum_{t}e_{i}^{2}(t)$, p1 and p2 are the number of parameters in the restricted and full models, respectively, and *m* is the number of sample points. If the value of $F_{statistic}$ is less than the value of the *F*-distribution with a confidence level of α, the null hypothesis of no causality can be rejected, indicating the presence of GC from $X_{j}$ to $X_{i}$.

### Appendix A.2. Improvements of DTF and PDC

The DTF measures the causal relationship between variables in the frequency domain. To further quantify DTF results and represent the strength of causal relations between variables as a matrix, Franaszczuk and Bergey proposed the integrated DTF (iDTF) [70]. The iDTF is calculated using Equation (A2), which sums the causal effects within a specific frequency band.

(A2) $$\begin{array}{c} {iDTF}_{ij}=\frac{1}{f_{2}-f_{1}}\sum_{f=f_{1}}^{f_{2}}\frac{H_{ij}\left(f\right)}{\sum_{k=1}^{n}\sqrt{{\left|H_{ik}\left(f\right)\right|}^{2}}} \end{array}$$

The integrated PDC (iPDC) was proposed by Astolfi et al. [71]. Similar to the iDTF, it is calculated by

(A3) $$\begin{array}{c} {iPDC}_{ij}=\frac{1}{f_{2}-f_{1}}\sum_{f=f_{1}}^{f_{2}}\frac{A_{ij}\left(f\right)}{\sum_{k=1}^{n}\sqrt{{\left|A_{kj}\left(f\right)\right|}^{2}}}. \end{array}$$

Korzeniewska et al. proposed the full-frequency DTF (ffDTF) [72]. It is calculated with Equation (A4), which normalizes the sum of causal effects from other variables to $X_{i}$ within a specified frequency band.

(A4) $$\begin{array}{c} {ffDTF}_{ij}=\frac{\sum_{f=f_{1}}^{f_{2}}H_{ij}\left(f\right)}{\sum_{k=1}^{n}\sum_{f^{\prime }=f_{1}}^{f_{2}}\sqrt{{\left|H_{ik}\left(f^{\prime }\right)\right|}^{2}}} \end{array}$$

To detect time-varying causality, Astolfi et al. proposed the adaptive DTF (ADTF) [73]. When the DTF between variables at certain frequencies is high and the signal’s spectral energy at that frequency is low, measurement of the DTF or PDC might be unreliable. To address this issue, Van Mierlo et al. proposed the spectrum-weighted DTF (swDTF) [74]. It is defined as

(A5) $$\begin{array}{c} {swDTF}_{ij}\left(t\right)=\frac{\sum_{f=f_{1}}^{f_{2}}{\left|H_{ij}\left(f,t\right)\right|}^{2}\sum_{k=1}^{K}{\left|H_{jk}\left(f,t\right)\right|}^{2}}{\sum_{l=1}^{K}\left(\sum_{f^{\prime }=f_{1}}^{f_{2}}{\left|H_{il}\left(f^{\prime },t\right)\right|}^{2}\sum_{s=1}^{K}{\left|H_{ls}\left(f^{\prime },t\right)\right|}^{2}\right)} \end{array}$$

where $\sum_{k=1}^{K}{\left|H_{jk}\left(f,t\right)\right|}^{2}$ and $\sum_{s=1}^{K}{\left|H_{ls}\left(f^{\prime },t\right)\right|}^{2}$ represents the power spectrum of variable $X_{j}$ and $X_{l}$, respectively.

The spectrum-weighted PDC (swPDC) was proposed by Plomp et al. [75], which is similar to swDTF and is calculated by

(A6) $$\begin{array}{c} {swPDC}_{ij}\left(f,t\right)=\frac{{\left|A_{ij}\left(f,t\right)\right|}^{2}}{\sum_{k=1}^{n}{\left|A_{kj}\left(f\right)\right|}^{2}}S_{j}\left(f,t\right) \end{array}$$

where $S_{j}(f,t)$ is the power spectrum of $X_{j}$.

### Appendix A.3. Calculation of TE

Transfer entropy, proposed by Schreiber, is based on the concept of information theory and Shannon entropy [65]. It reflects the flow of information from one variable to another. A higher transfer of information indicates a stronger causal relationship. In the definition of TE in Equation (21), $H\left(Y_{t+h}|Y_{t}\right)$ and $H\left(Y_{t+h}|X_{t},Y_{t}\right)$ are conditional entropies calculated by Equations (A7) and (A8). Here, p(·,·) represents the joint probability density function and $p\left(\cdot |\cdot \right)$ represents the conditional probability density function. ${\vec{x}}_{t}$ and ${\vec{y}}_{t}$ are the embedding vectors of *X* and *Y* at time *t*, and $l_{1}$ and $l_{2}$ are the dimensions of the embedding vectors ${\vec{x}}_{t}$ and ${\vec{y}}_{t}$, respectively. τ is the sampling interval of the embedding vector.

(A7) $$\begin{array}{c} H\left(Y_{t+h}|Y_{t}\right)=-\sum_{t}p\left({\vec{y}}_{t}\right)\log p\left({\vec{y}}_{t}\right)-\left(-\sum_{t}p\left({\vec{y}}_{t},y_{t+h}\right)\log p\left({\vec{y}}_{t},y_{t+h}\right)\right) \end{array}$$

(A8) $$\begin{array}{c} H\left(Y_{t+h}|X_{t},Y_{t}\right)=-\sum_{t}p\left({\vec{x}}_{t},{\vec{y}}_{t}\right)\log p\left({\vec{x}}_{t},{\vec{y}}_{t}\right)-\left(-\sum_{t}p\left({\vec{x}}_{t},{\vec{y}}_{t},y_{t+h}\right)\log p\left({\vec{x}}_{t},{\vec{y}}_{t},y_{t+h}\right)\right) \end{array}$$

According to Equations (A7) and (A8), $H\left(Y_{t+h}|Y_{t}\right)$ represents the uncertainty of $y_{t+h}$ given ${\vec{y}}_{t}$, and $H\left(Y_{t+h}|X_{t},Y_{t}\right)$ represents the uncertainty of $y_{t+h}$ given both ${\vec{x}}_{t}$ and ${\vec{y}}_{t}$. Therefore, TE quantifies the reduction of uncertainty of $y_{t+h}$ when the past values of variable *X* are introduced. The larger the $T_{X\to Y}$ is, the stronger the causal relationship from *X* to *Y* will be. Conversely, if $T_{X\to Y}$ is zero, there is no causal relationship from *X* to *Y.* By substituting the calculation formulas of conditional entropy into Equation (21) and simplifying it, the calculation formula of TE can be obtained, as shown in Equation (A9).

(A9) $$\begin{array}{c} T_{X\to Y}=\sum_{t}p\left(y_{t+h},{\vec{x}}_{t},{\vec{y}}_{t}\right)\cdot \log\frac{p\left(y_{t+h}|{\vec{x}}_{t},{\vec{y}}_{t}\right)}{p\left(y_{t+h}|{\vec{y}}_{t}\right)} \end{array}$$

### Appendix A.4. DTE

Information transferred from one variable to another may contain information from both itself and other variables, as illustrated in Figure A1. In the left figure, variable *X* has an effect on variable *Z,* and variable *Z* has an effect on variable *Y*. Consequently, some information of variable *X* may flow to variable *Y* through variable *Z*. In the right figure, both variables *X* and *Y* are influenced by variable *Z*, meaning that both variables contain information originating from *Z*. Both scenarios can lead to an overestimation of the TE between *X* and *Y* compared to the true situation.

To address the above issue, Duan et al. proposed direct transfer entropy (DTE) [2], which is defined as

(A10) $$\begin{array}{c} T_{\left.X\to Y\right|Z}=H\left(Y_{t+h}|Y_{t},Z_{t}\right)-H\left(Y_{t+h}|X_{t},Y_{t},Z_{t}\right) \end{array}$$

where $Z_{t}$ is the condition variable. DTE further quantifies the reduction of the uncertainty of *Y* given the past values of the condition variable *Z* after accounting for *X*, thereby eliminating the information of *Z* from the information transferred from *X* to *Y*.
